# Blood Lead Levels: Rhoads and Brown Respond

**DOI:** 10.1289/ehp.11636R

**Published:** 2008-11

**Authors:** George Rhoads, Mary Jean Brown

**Affiliations:** Advisory Committee on Childhood Lead Poisoning Prevention School of Public Health, University of Medicine and Dentistry of New Jersey, Piscataway, New Jersey; Lead Poisoning Prevention Branch Centers for Disease Control and Prevention, Atlanta, Georgia, E-mail: mjb5@cdc.gov

Rothenberg states that the level of concern for blood lead in children should be lowered from 10 μg/dL, but he provides little cogent rationale for doing so. As basic science support, he cites studies that *a*) exposed isolated neurons to a 3 μM solution of lead (Ishihara et al. 1995), a concentration that is 5,000 times the plasma levels expected in a child with a blood lead level (BLL) of 5 μg/dL (Manton et al. 2001); *b*) a study of squirrel monkeys exposed *in utero* to maternal BLLs in the 20- to 70-μg/dL range (Newland et al. 1996); and *c*) a study of occupationally exposed workers with a median BLL of 17.1 μg/dL that contains no data on neurologic effects of lead (Murata et al. 2003). Rothenberg’s choice of these citations emphasizes how little basic science work has been done on neuro-developmental effects at the very low levels of lead under discussion.

There was a 90% decrease in U.S. childhood BLLs from the late 1970s to the late 1990s (Pirkle et al. 1994). If the regression coefficients relating BLLs < 10 μg/dL to cognitive functions are taken at face value, they predict a population-wide, half standard deviation of cognitive improvement as a result of this fall in blood lead—a remarkable shift that should have substantially increased the number of very bright students with IQ > 135. To our knowledge, no such effect has been noted in the education literature, nor is it evidenced, for instance, among the increasing proportion of U.S. students admitted to U.S. graduate programs (Basken 2006).

What measures can be used to look more formally for this IQ improvement? IQ itself is problematic because the Flynn effect and adjustments in test instruments make secular changes in IQ hard to interpret. The teaching content in science and math has likely shifted over this period. Therefore, in our editorial (Brown and Rhoads 2008) we cited reading scores that measure a key skill that has been identified repeatedly as being affected by lead exposure among other factors. Campbell et al. (2000) reported that reading scores, examined with a suitable time lag in large nationally representative samples of children, were virtually unchanged over the critical period of declining lead levels. Rothenberg is correct that modest gains in math and science were recorded, but these changes could easily have other explanations, and they do nothing to explain the absence of any signal in reading scores. Although there may be other explanations for this absence, the simplest explanation of this paradox is that the published regression coefficients relating BLLs < 10 μg/dL to cognitive measures, all of which come from observational studies, are biased. This possibility is suggested by the steepening of the IQ curve at low lead levels [Centers for Disease Control and Prevention (CDC) 2005b].

Regardless of one’s view of the above evidence, it is important to recognize that virtually all of the progress made in eliminating childhood lead poisoning has been through primary prevention—the control or elimination of lead sources before children are exposed. This approach has lowered the proportion of 1- to 5-year-old children with BLLs > 10 μg/dL from well above 50% 30 years ago to 1.6% in 1999–2002 (CDC 2005a). The percentage is almost certainly lower today. Primary prevention has been proven to work and deserves the continuing attention that we described in our editorial (Brown and Rhoads 2008). Primary prevention can, and should, include increased attention to controlling exposures from lead paint hazards, imported foods, medicines, cosmetics, and toys. Renewed emphasis on screening with a lower BLL of concern would be expensive, intrusive to families, and hard to justify in the absence of proven, practical strategies for reducing lead levels in identified children. Further, it would likely deflect needed resources away from the primary prevention effort.

The CDC, in collaboration with federal, state, and local agencies, has outlined and begun to implement a comprehensive, society-wide effort to prevent lead exposure in children while maintaining efforts to identify and treat children with elevated BLLs (CDC 2005b). The CDC has also developed specific recommendations for health care and social service providers, scientists, and public health practitioners who are interested in actively participating in these primary prevention efforts by providing valuable leadership and expertise (Binns et al. 2007; CDC 2005b). By working together with federal, state, and local agencies to foster expansion of primary prevention services, these child advocates can accelerate achieving our mutual goal—lead-safe environments for the nation’s children.
